# The metastasis suppressor KISS1 is an intrinsically disordered protein slightly more extended than a random coil

**DOI:** 10.1371/journal.pone.0172507

**Published:** 2017-02-16

**Authors:** Alain Ibáñez de Opakua, Nekane Merino, Maider Villate, Tiago N. Cordeiro, Georgina Ormaza, Marta Sánchez-Carbayo, Tammo Diercks, Pau Bernadó, Francisco J. Blanco

**Affiliations:** 1 CIC bioGUNE, Derio, Spain; 2 Centre de Biochimie Structurale, INSERM U1054, CNRS UMR 5048, Université Montpellier 1 and 2, Montpellier, France; 3 Lucio Lascaray Research Center, Universidad del País Vasco, Vitoria, Spain; 4 IKERBASQUE, Basque Foundation for Science, Bilbao, Spain; University of Pittsburgh School of Medicine, UNITED STATES

## Abstract

The metastasis suppressor KISS1 is reported to be involved in the progression of several solid neoplasias, making it a promising molecular target for controlling their metastasis. The KISS1 sequence contains an N-terminal secretion signal and several dibasic sequences that are proposed to be the proteolytic cleavage sites. We present the first structural characterization of KISS1 by circular dichroism, multi-angle light scattering, small angle X-Ray scattering and NMR spectroscopy. An analysis of the KISS1 backbone NMR chemical shifts does not reveal any preferential conformation and deviation from a random coil ensemble. The backbone ^15^N transverse relaxation times indicate a mildly reduced mobility for two regions that are rich in bulky residues. The small angle X-ray scattering curve of KISS1 is likewise consistent with a predominantly random coil ensemble, although an ensemble optimization analysis indicates some preference for more extended conformations possibly due to positive charge repulsion between the abundant basic residues. Our results support the hypothesis that KISS1 mostly samples a random coil conformational space, which is consistent with its high susceptibility to proteolysis and the generation of Kisspeptin fragments.

## Introduction

Metastasis suppressors are able to slow or block metastasis without preventing primary tumor development. The *KISS1* gene was originally identified as a potent melanoma metastasis suppressor [1], where expression levels correlated inversely with the metastatic potential in a panel of melanoma cell lines [2]. The same inverse correlation with tumor stage and overall survival rate was also described for bladder cancer, where every tumor that had developed distant metastasis showed complete absence of *KISS1* expression [3, 4]. Similar observations on tumor progression, metastasis, and survival in different human cancers make KISS1 an interesting target for controlling metastasis in a therapeutic context [5]. Human KISS1 has an N-terminal secretion signal sequence of 19 residues, and secretion is essential for its metastasis suppression ability [6]. After secretion KISS is processed by the furin endoprotease [7]. This enzyme recognizes dibasic cleavage sites on the amino acid sequence and, after the action of carboxypeptidases and peptidyl-glycine-α-amidating monooxygenase (PAM), generates a fragment of 54 residues known as Kisspeptin54 (KISS1 residues 68–121, with an amidated C-terminus)[8]. Although furin can associate with the protein inside the cells it only cleaves KISS1 outside the cells [7]. Kisspeptin54 can be further cleaved into smaller 14, 13 and 10 residue fragments, or kisspeptins, which are ligands of the seven-transmembrane helix G protein-coupled receptor GPR54, also named KISS1R [9–11]. KISS1R regulates the secretion of gonadotropin releasing hormone from the hypothalamus, affects mammalian reproduction, and initiates puberty in a variety of species [12]. Kisspeptins act as neurotransmitters and neuroendocrine regulators [13], and have been implicated in hypogonadism [14] and Alzheimer’s disease [15]. Stimulation of KISS1R by exogeneous Kisspeptins releases intracellular calcium stores and also activates the MAP kinase pathway [9]. However, the role of KISS1R in metastasis is unclear since cell lines suppressed for metastasis by KISS1 do not express KISS1R, and KISS1 that cannot be processed by furin still suppresses metastasis, suggesting that autocrine signaling is not required for antimetastatic function [5] and that alternative signaling pathways exist [16].

While the structure of KISS1 is unknown, its amino acid sequence suggests that it is intrinsically disordered. Many proteins either lack secondary and/or tertiary structure entirely, or possess long unstructured segments under physiological conditions [17, 18]. They are collectively referred to as intrinsically disordered proteins (IDPs) and it is now widely recognized that IDPs play diverse biological roles [19]. For instance, most transcription factors [20] and proteins involved in cell signaling [21] in eukaryotes are predicted to be disordered or to contain long disordered segments. More than three-quarters of human cancer associated proteins have been classified as IDPs. In comparison, only about half of all eukaryotic proteins in the UNIPROT database are IDPs [21]. This observation underlines the importance of intrinsic disorder in the function of proteins regulating diverse processes that are often altered in cancer.

Due to the intrinsic high flexibility of IDPs, nuclear magnetic resonance (NMR) is the method of choice to study their conformational preferences [22]. Several NMR observables have been used to characterize IDPs [23], where chemical shifts and their deviation from random coil values are most widely used. Small-angle X-ray scattering (SAXS) has the capacity to report on the conformational space sampled by disordered states and therefore complements the local information provided by NMR [24, 25]. Integration of these experimental data into computational models aids in elucidating the structure-function relationships for this important, yet elusive class of proteins [26].

Here we present the first structural characterization of human KISS1. Circular dichroism (CD), SAXS, and NMR measurements (including chemical shifts and backbone ^15^N NMR transverse relaxation times) are consistent with a largely disordered protein without detectable local conformational preferences, but with a tendency to be more extended than a statistical coil. Our work paves the way to structural studies on the functional interactions of KISS1 with other proteins.

## Materials and methods

### Protein expression and purification

The gene encoding human KISS1 without the signal sequence and with codon usage optimized for bacterial expression cloned into a pET11d vector was purchased from Entelechon. Luria Broth containing 100 μg/mL ampicillin was inoculated with a clone of *E*. *coli* BL21(DE3) cells transformed with the KISS1-pET11d construct. Cells were grown at 37°C to an OD_600_ of 0.6 and protein expression was induced with 1 mM IPTG for 3 h at 37°C. For NMR studies, the cells from 3 L of LB were resuspended in 1 L of M9 minimal medium with isotopic enrichment (1 g/L 99% ^15^NH_4_Cl and 2 g/L 99% ^13^C_6_-glucose) and expression induced with 1 mM IPTG for 3 h at 37°C [27]. Cells were harvested and resuspended in lysis buffer (20 mM Tris, pH 8.0, 1 mM DTT, plus one tablet of Complete protease inhibitor cocktail), and sonicated on ice. The lysate was clarified by ultracentrifugation at 4°C and the supernatant was loaded on a Hiload 26/10 Q Sepharose column (GE Healthcare) with a column volume (CV) of 53 mL. The column was washed with 20 mM Tris, pH 8.0, 1 mM DTT, 1 mM EDTA (buffer A) and the protein was eluted with a linear gradient of salt up to 2 M NaCl in 3 CV). Fractions containing the protein (as seen in SDS-PAGE) were pooled and prepared for reverse phase chromatography by adding trifluoroacetic acid (TFA) to final concentrations of 0.1%. The solution was clarified by a 0.22 μm filter and loaded on a Phenomenex Jupiter C_18_ 250x10 mm column with 10 μm particles and 300 Å pore diameter previously equilibrated with 0.1% aqueous TFA. The protein was eluted with a 0–55% gradient of elution buffer (90% aqueous acetonitrile, 0.1% TFA) in 3 CV and freeze-dried. The purity of the sample was evaluated by overloaded SDS-PAGE analysis, and mass spectrometry measurements indicated that the initial methionine was processed by bacterial enzymes. For NMR analysis the lyophilized protein was dissolved in water with 2 mM DTT and the pH adjusted to 5.7. For other biophysical analysis the powder was dissolved in PBS (10 mM phosphate, 140 mM chloride, 153 mM sodium ion, 4.5 mM potassium ion) pH 7.0, 1 mM DTT, chromatographed on a HiLoad Superdex 200 16/60 column (GE Healthcare), and concentrated by ultrafiltration using Amicon devices with 3 kDa cut-off. Protein concentration was determined by UV absorbance at 280 nm using the extinction coefficient calculated from the amino acid composition (6990 M^-1^·cm^-1^).

### Size Exclusion Chromatography—Multi Angle Light Scattering (SEC-MALS)

These experiments were performed using a Superdex 200 10/300 GL column (GE HealthCare) connected to a DAWN-HELEOS light scattering detector and an Optilab rEX differential refractive index detector (Wyatt Technology) at 25°C. The column was equilibrated with running buffer (PBS pH 7.0, 1 mM TCEP, 0.03% NaN_3_) and the SEC-MALS system was calibrated with a sample of Bovine Serum Albumin (BSA) at 1 g/L in the same buffer. A sample of 100 μL of Kiss1 protein at 0.8 g/L was injected at 0.5 mL/min. Data acquisition and analysis employed ASTRA software (Wyatt). Based on numerous measurements on BSA samples under the same or similar conditions, we estimate that the experimental error in molar mass is around 5%.

### CD spectroscopy

The spectrum of a 52 μM sample of Kiss1 in PBS pH 7.0, 0.1 mM DTT was recorded on a Jasco-815 spectropolarimeter using a quartz cuvette (0.1 mm path length). Thermal denaturation was induced by heating (at a rate of 1°C/min) a sample of 8.0 μM Kiss1 in the same buffer in a stoppered cuvette with 2 mm path length.

### NMR spectroscopy

NMR experiments were recorded at 25°C on a Bruker Avance III spectrometer operating at 18.8 T (800.1 MHz ^1^H Larmor frequency) equipped with a TCI cryo-probe and z gradients. Spectra were processed with TopSpin (Bruker) and analyzed using Sparky [28]. ^1^H chemical shifts were referenced directly, ^13^C and ^15^N chemical shifts indirectly [29], to added 2,2-dimethyl-2-silapentane-5-sulfonate (DSS, methyl ^1^H signal at 0.00 ppm). The experiments for spectral assignment were obtained using a 112 μM [U-^13^C, ^15^N] KISS1 sample in water with 5% (v/v) ^2^H_2_O, 2 mM DTT and pH 5.7 adjusted with concentrated HCl and NaOH. A pH lower than 7.0 was chosen to reduce the solvent exchange of the amide protons that made not visible several of the Kiss1 signals in the ^1^H,^15^N-HSQC spectrum at pH 7.0 (data not shown). The value of 5.7 was considered appropriate as only the two histidine residues of KISS1 will be affected (becoming predominantly protonated in the NMR sample). ^1^H^N^, ^15^N, ^13^C′, ^13^C^α^, ^13^C^β^ and ^1^H^α^ assignments were obtained from the analysis of two dimensional ^1^H,^15^N-HSQC, ^1^H,^13^C-HSQC and three dimensional HNCO, HN(CA)CO, HNCACB, HN(CO)CACB, HNCA, HN(CO)CA, HN(CA)HA, and HN(COCA)HA experiments. A partial automatic assignment of the backbone and ^13^C^β^ resonances was obtained with the program MARS [30] and then completed manually. Several ^1^H^α^ assignments were obtained or confirmed in the ^1^H,^13^C HSQC spectrum. The assignment has been deposited in the BioMagResBank (accession number 26935). Secondary chemical shift values were calculated as the difference between the measured chemical shifts and their amino-acid specific random-coil values [31] using nearest-neighbor amino acid sequence corrections [32]. The latter were obtained from the web server http://www1.bio.ku.dk/english/research/bms/research/sbinlab/groups/mak/randomcoil/script/, which also includes corrections for temperature and pH effect [33]. Backbone amide ^15^N transverse (T_2_) relaxation times were measured at 25°C and 81.1 MHz ^15^N Larmor frequency. The pulse sequence for ^1^H detected ^15^N T_2_ relaxation measurement [34] was implemented in a series of two dimensional ^1^H-^15^N HSQC spectra with different ^15^N T_2_ relaxation delays (48.05, 64.06, 80.08, 128.13, 192.19, 256.26, and 400.4 ms). The HSQC experiments were acquired in an interleaved mode and the intensity of the backbone amide signals was fitted to a single exponential decay. Signal overlap prevented the reliable measurement of the signal intensity for a number of residues, while the C-terminus yielded an anomalously high T_2_ value with a very large error and was excluded from the analysis.

### Small-Angle X-ray Scattering (SAXS) measurement and analysis

SAXS measurements were carried out at the BM-29 BioSAXS beamline at the ESRF Storage Ring (Grenoble, France) using a sample-to-detector distance of 2.87 m [35]. KISS1 samples were measured at 10°C (to minimize radiation damage) and concentrations of 4.0 and 1.0 g/L in PBS, pH 7.0, 1 mM DTT. The scattering pattern of the buffer solution was recorded before and after measuring each protein sample. The obtained scattering profiles covered a momentum transfer range of 0.003 < *s* < 0.49 Å^-1^. A comparison of ten consecutive X-ray exposures indicated the absence of radiation damage. The final curves at each concentration were derived after subtracting the averaged buffer from the protein patterns using standard protocols within PRIMUS [36]. SAXS curves at both concentrations were merged to derive a final profile that was used for the subsequent structural analysis. The forward scattering, *I(0)*, and the radius of gyration, *R*_*g*_, were evaluated using Guinier's approximation [37], assuming that at very small angles (*s* < 1.3/*R*_*g*_), the intensity can be described as I(*s*) = *I(0)* exp(−(*sR*_*g*_)^2^/3). The pairwise distance distribution function, *p(r)*, and the maximum particle diameter, *D*_*max*_, were computed with GNOM [38] using a momentum transfer range of 0.013 < *s* < 0.29 Å^-1^.

The ensemble optimization method (EOM) was used to describe the overall properties of KISS1 in solution [24]. To this aim a pool of 10,000 conformations was built with Flexible-Meccano [39, 40] and side-chains were added with Sccomp [41]. For each conformation the theoretical SAXS curve was computed with Crysol [42] and used for the EOM sub-ensemble optimization. Two hundred independent EOM runs with 50 selected conformers were performed using the χ^2^ statistical test as the selection criterion. The resulting ensemble composed of 10,000 conformations was structurally evaluated using its *R*_*g*_ distribution.

## Results

### Sequence analysis and disorder prediction

**Fig 1:**
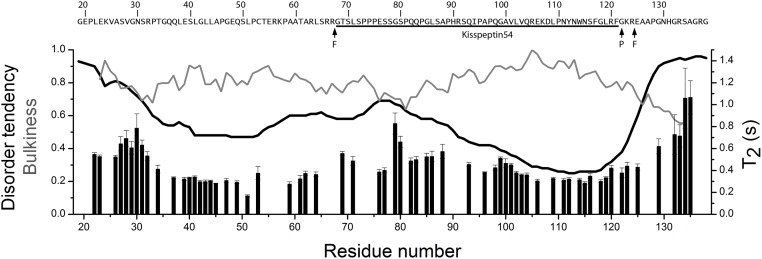
Amino acid sequence features and backbone dynamics of KISS1. The sites of proteolytic processing by furin are indicated with arrows F. The site of peptidyl-glycine-α-amidating monooxygenase (PAM) action that converts G122 into an amide is indicated by arrow P. The black line shows the predicted disorder tendency (left axis), the grey line shows the normalized bulkiness along the polypeptide chain (left axis), and the bars show the backbone amide ^15^N T_2_ relaxation times (right axis). The bulkiness was calculated with the ProtScale tool in the expasy web server (http://web.expasy.org/protscale/) with a window size of 9.

The amino acid composition of KISS1 shows a high content of polar residues (more than 60%, a feature that is typical for intrinsically disordered proteins [43]. The degree of disorder in KISS1 was predicted with the metaPrDOS web server that integrates the results of eight different methods [44]. Regions with a disorder tendency smaller than 0.5 comprise residues 41–53 and 91–123 (Fig 1). The latter region with reduced disorder tendency is largely contained within Kisspeptin54, the major fragment of KISS1 that encompasses residues 68–121. A prediction of protein binding regions with the web server ANCHOR [45] identified two long segments (18–71 and 84–127) that contain both regions with reduced disorder tendency.

### Protein sample preparation

The polypeptide studied here corresponds to the full length KISS1 amino acid sequence (as described in Uniprot entry Q15726) without the secretion signal peptide, and encompass KISS1 residues 19–138 (with a theoretical molecular weight of 12.6 kDa). No extraneous residues that could perturb the native structural features of the protein were introduced. Since no affinity tag was used, high expression levels of the recombinant gene were necessary for efficient protein purification. Considering that the human cDNA sequence coded two leucine residues with codons of low abundance in *E*. *coli*, a synthetic gene with codons optimized for bacterial expression was used. KISS1 was then purified by anion exchange, reverse phase, and gel filtration chromatographic separations. Still the yield of pure protein was very low, between 0.1 and 0.6 mg/L of bacterial culture.

### Structural analysis by SEC-MALS and CD

**Fig 2:**
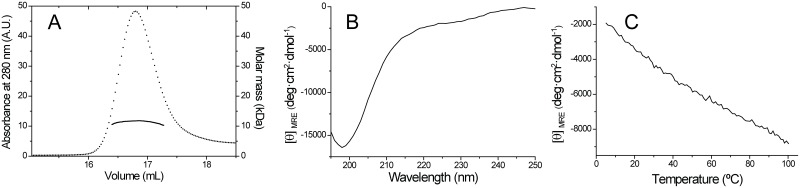
KISS1 is monomeric and has little secondary and no tertiary structure in solution. (A) Size exclusion chromatogram of KISS1 (thin line and axis to the left), and molar mass derived from MALS (thick line and axis to the right). (B) CD spectrum of KISS1 at 25°C. (C) Thermal denaturation as measured by changes in the mean residue molar ellipticity at 222 nm.

The protein eluted from the SEC column at a volume corresponding to an apparent molar mass of 28 kDa, according to calibration with molecular weight standards. The mass derived from MALS data is 11.7 kDa (Fig 2A), however, demonstrating that Kiss1 is monomeric. These inconsistent results indicate that Kiss1 is either folded with an elongated shape, or flexible and disordered [46]. In either case it would elute at a smaller volume than a globular protein of the same mass.

The far-UV CD spectrum of Kiss1 shows a minimum at 198 nm and a shoulder at 226 nm, consistent with a predominantly random-coil protein with little secondary structure (Fig 2B). The thermal denaturation curve of Kiss1 followed by the changes in the CD signal at 222 nm (Fig 2C) does not show any cooperative folding-unfolding transition, indicating that KISS1 lacks a defined tertiary structure.

### NMR assignment and analysis

**Fig 3:**
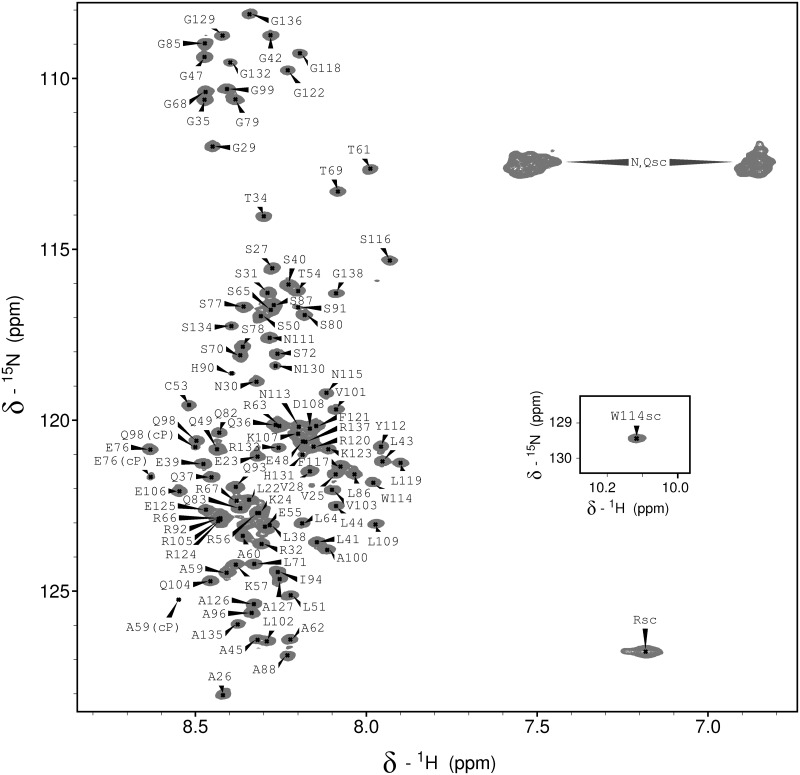
Residue-specific assignment of the ^1^H-^15^N HSQC NMR spectrum of KISS1. The signal pairs from the unassigned asparagine and glutamine side chain amide groups are labeled as N,Qsc. The unassigned signal from arginine side chain HE-NE groups is labeled as Rsc, and the inset shows the W114 indole signal. Minor signals that likely arise from cis-Pro isomers are labeled with “cP”.

The resonances of the KISS1 backbone and side chain C^β^ nuclei were assigned from the standard suite of 3D triple resonance spectra. For the N-terminal residues G19 and E20 no signals could be observed in the ^1^H-^15^N HSQC spectrum. For the remaining 103 non-proline residues the ^1^H-^15^N HSQC signals could be unambiguously assigned, as shown in Fig 3. This spectrum also shows a few minor signals with about 5% of the major signals' intensity. As some of them appear to be duplicate signals of residues preceded by a proline we suggest that they arise from small populations of KISS1 with *cis* isomeric prolyl peptide bonds. This suggestion is further supported by the ^13^C^β^ chemical shifts of the corresponding proline residues, which are close to the average value measured for cis prolines in proteins [47].

The low dispersion of the backbone ^1^H^N^ chemical shifts observed in the HSQC spectrum indicates that KISS1 is largely disordered and flexible under native conditions. Yet, two regions of KISS1 around residues 40 and 115 show relatively short transverse relaxation time T_2_ values (Fig 1) and correspond to sequences with reduced predicted disorder tendency. A shorter than average ^15^N T_2_ relaxation time results from restricted local flexibility on a fast (ns to ps) timescale, pointing to a local deviation from the otherwise unrestricted and uncorrelated mobility characteristic for IDPs. There is, however, some inverse correlation between local residue bulkiness and ^15^N T_2_ relaxation times (Fig 1), suggesting that local restrictions in polypeptide chain dynamics could also be explained by steric hindrance between side chains and the backbone, as found for other IDPs [48].

**Fig 4:**
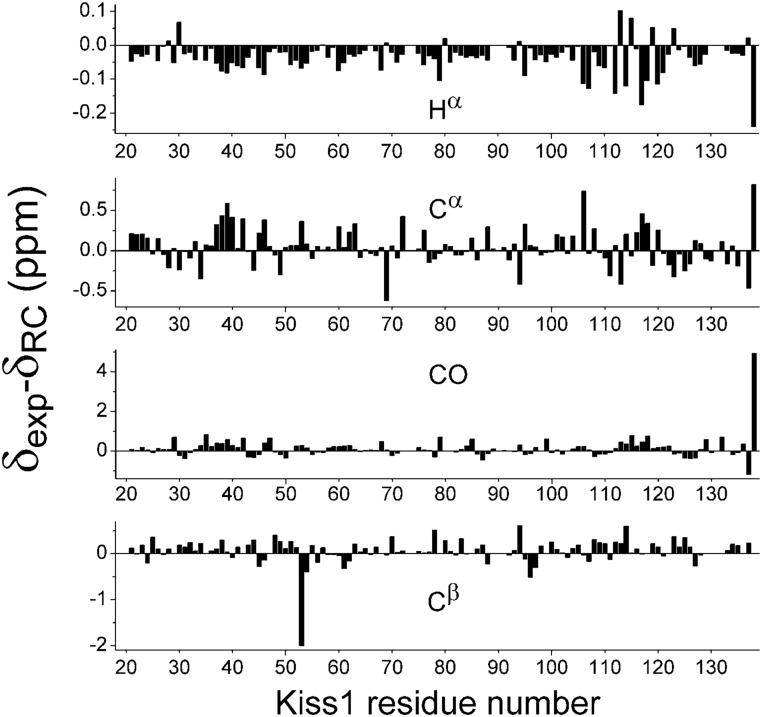
KISS1 residue plots of secondary chemical shifts for backbone and C^β^ nuclei referenced against their random coil values [33]. The large deviation of C^β^ for C53 is not due to cisteine oxidation. The measured value of 27.89 ppm indicates a reduced side chain [51].

The NMR chemical shift deviations were computed with respect to random coil values obtained from small disordered peptides [31]. Large deviations were observed only for the nuclei of the C-terminal residues and for the C^β^ of C53 (Fig 4). These deviations may derive from imperfection in the reference data set of random coil values, as noted before [49], rather than to local conformational preferences. For all other residues the chemical shift differences are within the RMSD measured in IDPs [50]. While the H^α^, C′, C^α^ shift deviations of residues 34 to 42 might suggest a local tendency for helicity, the values are very small and the tendency is not confirmed by the C^β^ shift deviations. In summary, our analysis of chemical shift deviations and ^15^N T_2_ relaxation times does not provide clear evidence for any conformational preference (Fig 4), suggesting that KISS1 behaves as a random coil polypeptide. However the ensemble of local conformations at the protonated histidine side chains might be different at pH 5.7 than at 7.0, used for the other measurements.

### SAXS analysis

**Fig 5:**
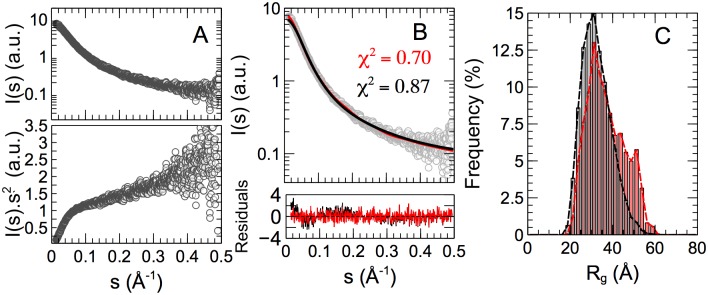
SAXS data and analysis on KISS1. (A) Logarithmic (top) and Kratky (bottom) representations of SAXS intensity versus momentum transfer *s* (open circles). (B) Averaged back-calculated curves derived from a statistical random coil ensemble (black line) and EOM selected sub-ensemble (red line). The goodness-of-fit is indicated by χ^2^ values. The bottom panel shows the residuals of the fitting for both conformational ensembles. (C) Distribution of the radius of gyration, Rg, in the random coil ensemble (gray-bars) and EOM selected sub-ensemble, where a shoulder peak around 50 Å contains ca. 30% of all conformers.

We furthermore collected SAXS data in order to probe the overall properties of KISS1 in solution [52]. Yet, the SAXS curve shown in Fig 5A presents no distinct features, and the Kratky representation is typical for a disordered protein, with a monotonic increase of I(*s*)*s*^2^ with the momentum transfer *s*. We observed a concentration effect on the SAXS data at the highest concentration used, and we merged curves measured at 1 and 4 mg/mL to reduce interparticle interactions while preserving a good signal to noise ratio. We used the Scåtter program [53] to calculate the molecular weight of the particles with a result of 9 kDa, indicating that the SAXS data correspond with a monomeric protein. An analysis of the smallest angle data by Guinier’s approach for a momentum transfer range *s·R*_*g*_ < 1.3 (where *R*_*g*_ is the radius of gyration) indicates that KISS1 has an average radius of gyration of 34.7 ± 0.5 Å. This value is slightly larger than expected for an IDP of 120 residues (R_g_^RC^ = 30.1 Å) suggesting that KISS1 could transiently adopt more extended structures [54].

In order to glean information on the conformational space sampled by KISS1 in solution from our SAXS data we modeled an atomistic ensemble of 10,000 KISS1 conformers using Flexible-Meccano [39], where torsion angle pairs were selected randomly from a database of amino acid specific conformations in loop regions of high-resolution X-ray structures [40]. After adding side-chains, the theoretical SAXS profile for each generated conformation was computed and then averaged over the entire ensemble. The resulting curve (Fig 5B), which represents a canonical random coil, agrees well with the experimental SAXS curve for KISS1 (χ_i_^2^ = 0.87). Yet, some systematic deviations are observed for small momentum transfer *s* ≤ 0.2 Å^-1^. A more detailed picture of the molecular sizes and shapes adopted by KISS1 in solution can be obtained via the Ensemble Optimization Method (EOM) [24] where a genetic algorithm selects a sub-ensemble of conformations that describes the experimental curve better than a random coil model (Fig 5B). EOM application to the SAXS curve of KISS1 yields a set of conformations that cover a broad range of molecular sizes, with a radius of gyration between 20 and 60 Å, consistent with a high level of disorder and flexibility (Fig 5C). Compared to the *R*_*g*_ distribution within the initial pool of conformations, which represents the canonical disorder of KISS1, the subset selected by EOM contains more conformations with larger *R*_*g*_ values. This result indicates a preference for extended conformations of KISS1 in solution.

## Discussion

The human tumor suppressor protein KISS1 is monomeric in solution, as shown by our SEC-MALS data, and CD measurements indicate no tertiary structure and little, if any, secondary structure. Thus, as found for many human cancer related proteins and as suggested also by its amino acid sequence, human KISS1 is an intrinsically disordered protein. The vast conformational heterogeneity of the chain makes it challenging to probe for local conformational preferences since all experimental data is ensemble averaged [55]. Nevertheless, we have been able to glean insight at the residue level by NMR. An analysis of chemical shifts did not consistently indicate regions with preferential conformations since differences with random coil values are small and within the range of the RMSD observed in IDPs [50]. It has been reported that Kisspeptin13 (residues 109–121) adopts helical structure in SDS micelles; this KISS1 fragment is a pharmacophore that served to identify hits with submicromolar affinity for the metastin receptor [56]. For full length KISS1 in aqueous solution, however, we do not find evidence for any preferential helical conformation in the same region. The same holds for the region corresponding to Kisspeptin54, which largely shows a random coil behavior as for the rest of the molecule. This is consistent with previously reported data on the isolated Kisspeptin54 fragment, for which a 3.7% total helical content was observed by CD but no distinguishable structural elements were detected by NMR [57]. The other sampled NMR parameter, ^15^N T_2_ relaxation times, does show some local deviations; however, the local reduction of flexibility appears to be due to sequence specific steric obstruction, rather than conformational rigidity. Overall, our NMR analysis of KISS1 indicates random coil behavior without local conformational preferences. A global tendency to populate more extended conformations, with a larger radius of gyration than expected for a random coil is suggested by our analysis of the SAXS data analysis. This tendency may be favored to mitigate positive charge repulsion between the abundant basic residues within the KISS1 sequence.

The functional role of IDPs presumably derives from their conformational versatility, allowing for interactions with different ligands, and for regulation through post-translational modifications at accessible sites [58]. In the case of KISS1, apart from phosphorylation at residue Y110 [59], the major modification is proteolysis by furin and C-terminal amidation by peptidyl-glycine-α-amidating monooxygenase [7]. KISS1 and its fragments interact with KISS1R, their GPCR receptor [9], but the mode of binding is unknown. Intrinsically disordered proteins may adopt folded or partially folded conformations upon binding to their partners [60]. This may occur via *i)* “induced fit” if the binding partner induces the disordered protein to adopt the conformation of the bound state, *ii)* “conformational selection” if the binding partner selects a favorable conformation from the ensemble present in the free state, or *iii)* a mixture of both mechanisms [61–63]. In the case of KISS1, the induced fit mechanism might be the most likely one for binding to KISS1R since there appears to be no preferred conformation in the solution state ensemble, although the situation might be different in the vicinity of the cell membrane. KISS1 has also been implicated in enhanced mitochondrial biogenesis by increasing the levels of peroxisome proliferator-activated receptor-γ co-activator 1-α (PGC1α), a transcriptional activator for many genes regulating mitochondrial mass and metabolism. The link between KISS1 and PGC1α might be the KISS1 interaction with ubiquilin-1 that protects PGC1α from degradation. However, the structural basis of the KISS1 interaction with ubiquilin-1 is unknown. Our work on full-length KISS1 lays the basis for further structural studies on its interactions with physiological partners.
